# Preclinical Alzheimer's and vascular biomarkers alter brain aging in cognitively normal adults: a MRI-based study

**DOI:** 10.3389/fnagi.2025.1653074

**Published:** 2025-11-24

**Authors:** Unai Torres, David Bernal, Ibai Gurrutxaga, Ainara Estanga, Pablo Martínez-Lage, Olatz Arbelaitz

**Affiliations:** 1 Department of Computer Architecture and Technology, University of the Basque Country UPV/EHU, Donostia, Spain; 2 Center for Research and Memory Clinic, CITA-Alzheimer Foundation, Donostia-San Sebastián, Spain

**Keywords:** brain aging, preclinical biomarkers, machine learning, kernel regression, Alzheimer's disease, vascular pathology

## Abstract

**Introduction:**

The aging global population underscores the need to understand brain aging and its links to neurodegenerative diseases. While most brain aging studies use cognitive impairment as exclusion criteria, preclinical biomarkers may influence results, potentially masking early pathological effects. This study evaluates how preclinical AD and vascular biomarkers impact brain aging models in cognitively normal subjects.

**Methods:**

Using baseline data from the European Prevention of Alzheimer's Dementia Longitudinal Cohort Study (EPAD LCS), we analyzed 1,380 cognitively unimpaired participants (50+ years) stratified into five groups based on cerebrospinal fluid biomarkers (Aβ42, t-tau, p-tau) and vascular pathology (Fazekas scale, microbleeds). Structural MRI volumes of cortical/subcortical regions were normalized and compared using Nadaraya-Watson kernel regression. Bootstrapping and Bonferroni-corrected statistical tests assessed differences in the relationship between age and brain volume between groups.

**Results:**

Significant differences emerged in the relationship between age and brain volume in biomarker-negative and biomarker-positive groups, particularly in the entorhinal cortex, amygdalas, and basal forebrain (*p* < 0.01). The AD and mixed AD/vascular groups showed the largest deviations. Gender-specific analyses revealed stronger effects in males. Vascular pathology alone affected distinct regions (e.g., left entorhinal cortex) without amygdala involvement, suggesting disease-specific atrophy patterns.

**Discussion:**

Preclinical AD and vascular biomarkers significantly alter brain aging in cognitively normal individuals. These findings highlight the importance of biomarker stratification in brain age studies to avoid biased estimates. Entorhinal cortex and amygdala volumes may serve as sensitive early indicators of neurodegeneration, supporting their use in targeted interventions and personalized monitoring.

## 1 Introduction

The global population is undergoing a rapid process of aging. Projections indicate that by the mid-2030s the number of people aged 65 years and over will reach 2.2 billion (United Nations, 2024). This demographic shift is accompanied by an increase in age-related diseases, particularly those affecting the brain (Seiler and Enzinger, 2025). As life expectancy continues to increase, research into the complexities of brain aging has become a critical area of study. The findings of such research have the potential to have far-reaching implications for society.

Brain aging is a multifaceted process involving structural, chemical and functional changes (Pacheco et al., 2015; Feng et al., 2020; Woodward et al., 2024). These alterations have the potential to induce cognitive decline, manifesting in impaired memory, diminished processing speed, and compromised executive functions. Although some degree of cognitive change is normal in the process of aging, the risk of neurodegenerative diseases, such as Alzheimer's and vascular conditions, also increases with age.

A more profound comprehension of cerebral senescence, encompassing both healthy individuals and those afflicted with diseases, stands to benefit society in myriad ways. It has the potential to inform the development of targeted interventions aimed at preserving cognitive health and postponing the onset of age-related brain disorders. This knowledge could inform public health strategies, potentially reducing the economic and social burden of caring for an aging population.

A significant endeavor has been undertaken in the domain of brain aging research, yielding encouraging results. Typically, this type of research involves the participation of healthy individuals who do not exhibit cognitive decline attributable to non-age-related factors. Nonetheless, it is acknowledged that neurodegenerative diseases, including Alzheimer's disease, and vascular pathologies initiate their effects on brain tissue well in advance of the manifestation of initial symptoms (Chételat et al., 2012; Doré et al., 2013; Bernal et al., 2024; Koenig et al., 2022).

Consequently, a number of studies were conducted to ascertain how incipient stages of neurodegenerative diseases, principally Alzheimer's disease, affect models that predict brain age based on cognitively normal subjects. In one of the earliest examples, Ly et al. (2020) noted that cognitively normal subjects can suffer from latent conditions (in particular, Alzheimer's disease) that affect brain aging years before the onset of cognitive impairment. It was asserted that their work constitutes a pioneering endeavor in the field, as it was the first to train a model to predict brain age by excluding subjects with amyloid plaques, as detected by PET imaging.

In subsequent years, there was a paucity of authors who went further in assessing the effect of such latent conditions on predictive models of brain age. In an attempt to analyze disparities in brain aging among subjects exhibiting varying stages of Alzheimer's disease, Hwang et al. (2022) conducted a study. The groups compared included healthy subjects, excluding those with pathological beta-amyloid protein values, and their results showed that machine learning models accurately separated age-related atrophy from disease-related neurodegeneration. In a similar vein, the extensive research of Cumplido-Mayoral et al. (2023a,b, 2024) sought to ascertain the factors that predominantly contribute to the disparities between predicted and actual brain age (Brain Age Gap or BAG). These factors encompass biomarkers, such as beta-amyloid and tau proteins, which are associated with Alzheimer's disease. Similarly, Marseglia et al. (2025) sought to identify associations between BAG and other factors, including life exposures, biological processes, and brain injury related to neurodegenerative and vascular disease (including AD and cerebrovascular neuroimaging markers). The findings of this study indicated that regular physical activity and better metabolic health were linked to lower BAG.

Despite the fact that such studies were capable of detecting effects on brain aging in patients in the early stages of neurodegenerative diseases, the majority of studies related to brain aging still utilize cognitive normality as their inclusion criterion, thereby overlooking potential preclinical conditions. It is acknowledged that, despite the high prevalence of certain diseases (e.g. Alzheimer's disease), these studies assume that the numerical superiority of subjects who are entirely healthy masks the effect of those affected.

For instance, the study by Guan et al. (2024) analyzed the effect of multimodal MRI features in the prediction of age, whilst the study by Dartora et al. (2024) utilized deep networks (in particular, convolutional networks) to predict brain age using barely processed MRI images. In addition, the study by Wittens et al. (2024) clinically evaluated a new brain age model on real-world clinical data and MRI scans of subjects across the entire AD continuum. It is notable that none of the aforementioned studies analyzed the potential latent conditions in the subjects of the healthy control group, such as abnormal protein levels (in particular, beta-amyloid or tau proteins) or vascular conditions. A similar phenomenon is observed in the work of Dörfel et al. (2023), wherein six libraries dedicated to brain age prediction were compared. Years earlier Elliott (2020) reviewed potential MRI-based biomarkers that could link midlife brain aging to later life dementia, but the aforementioned brain conditions were also ignored. Finally, a recent paper (Dular and piclin, 2024) proposed a standardized framework for evaluating brain age prediction models, which also obviates these concerns.

This summary demonstrates that, despite the presence of indications that biomarkers, such as beta-amyloid or p-tau, may influence brain aging, the majority of published studies have not yet considered this effect. The selection of healthy patients is predominantly predicated on cognitive or neurological assessments. Consequently, it is imperative to develop an analysis that examines the impact of these biomarkers on brain aging in cognitively healthy patients, to facilitate future research that can respond accordingly.

The present study aims to evaluate the impact of subjects who are not cognitively impaired but are in preclinical stages, on the results obtained in brain aging studies. The study focuses on biomarkers associated with Alzheimer's disease, the most prevalent neurodegenerative disease. Furthermore, the effects of vascular concerns, including microbleeds and strokes, are also studied.

The primary hypothesis of this study was that the results concerning brain aging, as determined from cognitively normal subjects, exhibit variation depending on the inclusion of subjects with positive biomarkers. In order to evaluate this hypothesis, the relation between age and volume of multiple brain regions was modeled in five subject groups, and the results obtained were compared. Furthermore, the results were analyzed in consideration of the subjects' gender and the statistical significance of the observed differences was assessed.

## 2 Materials and methods

The present study utilized baseline data from the European Prevention of Alzheimer's Dementia Longitudinal Cohort Study (EPAD LCS) (Ritchie et al., 2016; Solomon et al., 2018). The EPAD LCS is a prospective, multicentre, pan-European study designed to develop accurate longitudinal models that capture the full continuum of Alzheimer's disease progression and to establish a well-characterized cohort of individuals eligible for future enrollment in the EPAD proof-of-concept (PoC) clinical trials. Participants were eligible for inclusion in the study if they were 50 years of age or older, in generally good health, cognitively unimpaired at baseline, and had a study partner. The additional inclusion criteria demanded the capacity to undergo both MRI and lumbar puncture procedures. At the commencement of the study, participants underwent a thorough baseline assessment, after which they were followed up at six-month intervals for a period of three years.

Following the EPAD procedures, subsequent to the completion of a data access request through the ADDI Workbench, a platform developed by the Alzheimer's Disease Data Initiative (ADDI), we gained access to the EPAD LCS Version.IMI (V.IMI) dataset, which comprises the final longitudinal data with cognitive, clinical, biomarker, and neuroimaging and lifestyle risk factor datasets from the over 2,000 participants of the EPAD LCS. For the present cross-sectional study, the baseline data was utilized.

### 2.1 MRI data

The neuroimaging data in the EPAD LCS were acquired in accordance with a standardized protocol, as delineated by Solomon et al. (2018). The present study focused on quantitative volumetric measures derived from structural MRI. Specifically, we analyzed brain volume data from various cortical and subcortical regions, as these are considered to be among the most robust neuroimaging biomarkers of neurodegenerative changes in the preclinical and prodromal stages of Alzheimer's disease. In the EPAD study, structural MRI data were processed using the Lesion Explorer and Anatomical Parcellation (LEAP) pipeline, which facilitates automated segmentation and quantification of cortical and subcortical structures.

Brain volumes were processed in the course of the study. Initially, the data were normalized by dividing each volume by the subject's total intracranial volume (TIV), thus correcting for individual differences in skull size and shape. Following this, the data were standardized using z-score scaling.

The cerebrovascular burden was assessed using the Fazekas scale and the presence of cerebral microhaemorrhages. Participants were deemed to have a substantial cerebrovascular burden if they exhibited a Fazekas score of ≥2 or ≥4 microbleeds. The presence of brain lacunes, infarcts, and other relevant radiological findings was also evaluated as part of the eligibility assessment for study inclusion.

### 2.2 CSF biomarkers

Cerebrospinal fluid (CSF) samples were collected in accordance with a harmonized preclinical EPAD LCS protocol, and all analyses were conducted using the fully automated Roche Elecsys platform at a single certified laboratory (University of Gothenburg) to ensure analytical consistency. CSF biomarkers relevant to Alzheimer's disease including beta-amyloid (Aβ42), total tau (t-tau), and phosphorylated tau (p-tau), were quantitatively analyzed.

In the present study, the resulting biomarker values were utilized to classify participants according to their pathological status. The following thresholds were applied in order to define abnormal biomarker profiles:

- Aβ42: positive if < 1030 pg/mL
- t-tau: positive if > 300 pg/mL
- p-tau: positive if > 27 pg/mL
- p-tau/Aβ42 ratio: positive if > 0.023

**Figure 1:**
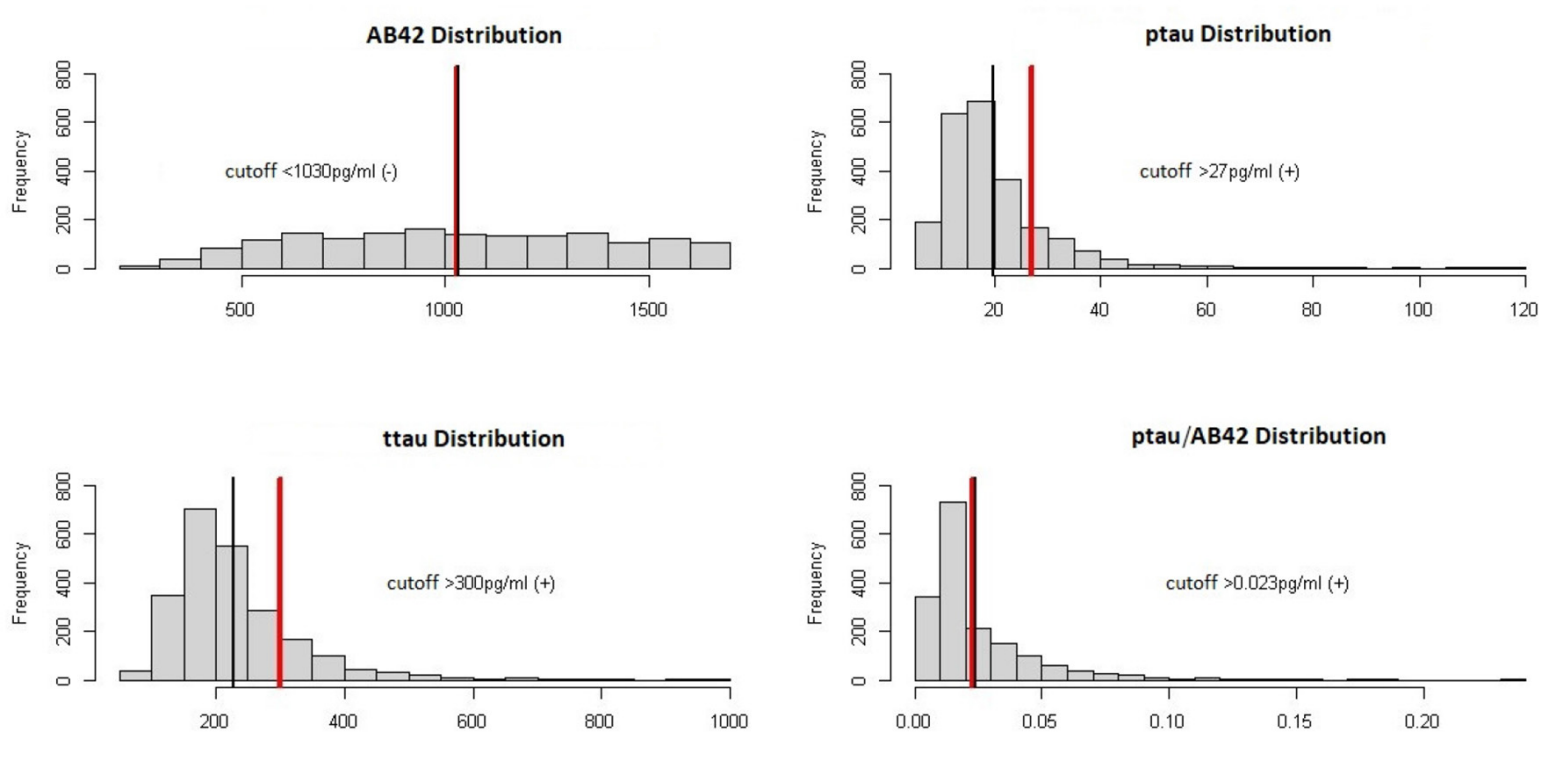
Biomarker distribution in the dataset. The black vertical line shows the median while the red vertical line shows the positive/negative threshold.

Figure 1 illustrates the distribution of these biomarkers.

### 2.3 Participants

#### 2.3.1 Inclusion and exclusion criteria

Participants included in this study were selected from the baseline visit of the EPAD LCS. The inclusion criteria comprised the following: The availability of CSF biomarker data (Aβ42, total tau, and phosphorylated tau) was the first criterion. The availability of high-quality structural MRI volumetric data was the second criterion. The quality of the volumetric data was assessed by excluding subjects that failed any of the WBLEAP QC Grade (LBVQC), LEAP Hippocampus QC Grade (LHQCG), LEAP Ventricular Volume QC Grade (LVVQC) and PTIV Factor QC Grade (PTIVQC) tests included in the dataset. The third criterion was the absence of cognitive impairment, defined as a global Clinical Dementia Rating (CDR) score of 0.

Participants were excluded if (1) radiological image readings revealed clinically relevant findings, including superficial siderosis, amyloid-related imaging abnormalities-edema (ARIA-E), or other abnormalities; and (2) they presented a vascular pathology profile (V+) combined with elevated phosphorylated tau (T+) but normal amyloid levels (A-). A total of 45 individuals were removed for this reason. After the implementation of the preprocessing and outlier removal, the dataset size was reduced to 1,397 examples. While this reduced the sample size to a considerable extent, it enhanced the quality of the data by removing problematic cases.

#### 2.3.2 Study groups

The subjects in the dataset were divided into five groups according to their biomarkers (Jack et al., 2024; Dubois et al., 2024; Agarwal et al., 2023; Duering et al., 2023; Wardlaw et al., 2013). To that end, four criteria were defined:

- A (Alzheimer's markers): Positive if Aβ42 < 1030 or p-tau/Aβ42 > 0.023.
- T (Phosphorylated tau protein marker): Positive if p-tau > 27.
- N (Total tau protein marker): Positive if t-tau > 300.
- V (Vascular pathology markers): Positive if Fazekas score was greater than 1, the presence of more than three microbleeds, or a history of stroke.

According to these four criteria five study groups were defined:

1. Normal group (N): All markers exhibited a negative response. Individuals were in good health and did not have any significant neurological issues. This group served as a reference for brain volume changes observed in other groups.
2. Alzheimer's Disease group (AD): subjects who were found to be Alzheimer's positive (A+), vascular negative (V-), with any T or N values. Individuals exhibiting Alzheimer's biomarkers, such as decreased Aβ42 protein and increased p-tau/Aβ42 ratio.
3. Vascular group (V): subjects who were found to be Vascular positive (V+), Alzheimer (A-) and tau negative (T-), with any N value. Patients with vascular brain conditions, but without Alzheimer's markers.
4. Alzheimer's Disease and Vascular group (AV): subjects who were found to be both Alzheimer's (A+) and Vascular positive (V+), with any T and N value. Patients with both Alzheimer's and vascular pathology markers, to study how the combination of these conditions affects brain volume.
5. Other Pathologies group (OP): subjects who were found to be Alzheimer's (A-) and vascular (V-) negative, but positive for either tau or total tau protein. Patients without Alzheimer's Disease or vascular markers but showing abnormal p-tau or t-tau biomarker levels.

A subset of 17 cases, characterized by an ambiguous combination of markers (A-, T+, V+, N any), was excluded from the study, resulting in a final sample size of 1,380 subjects.

**Figure 2:**
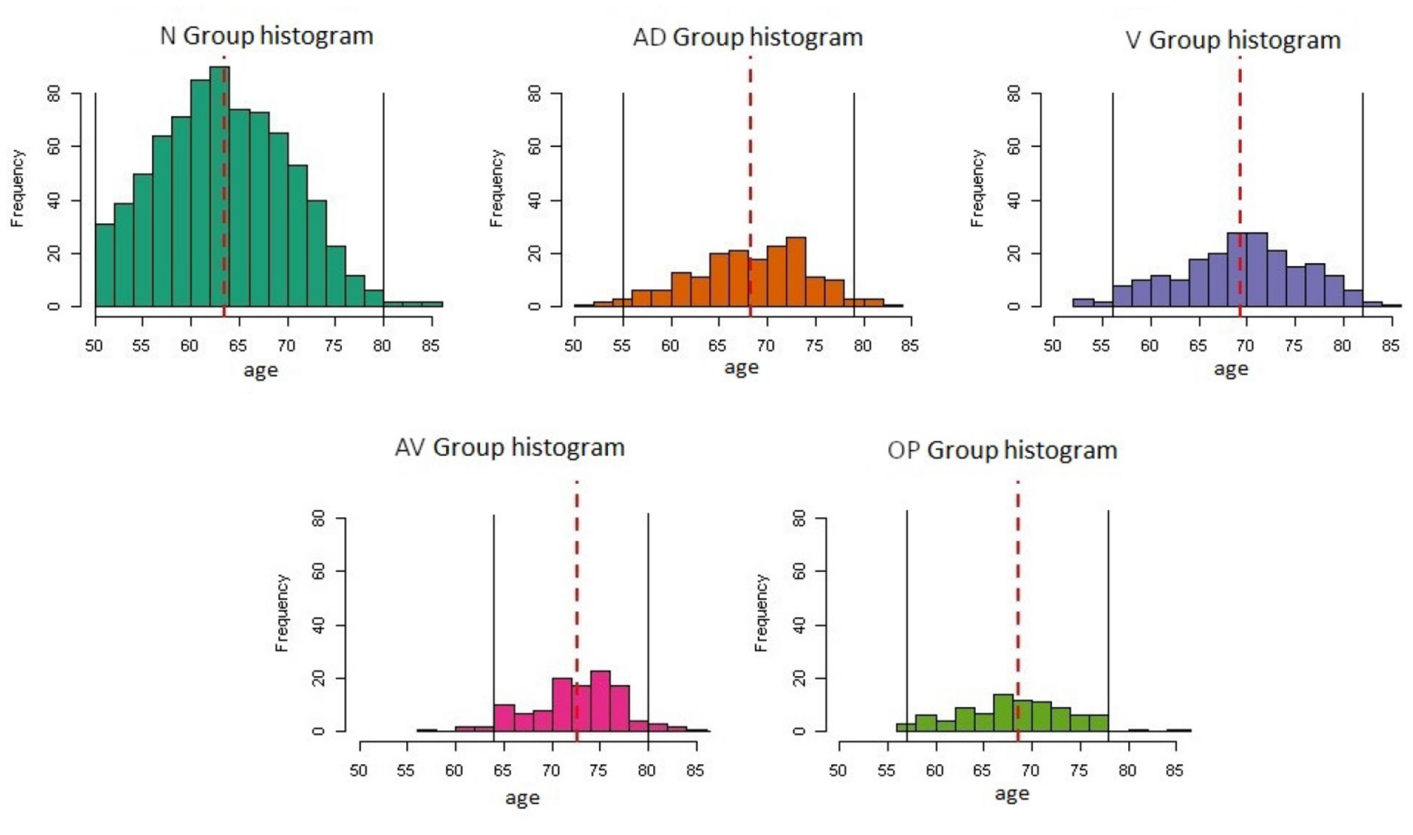
Histogram distribution of the different study groups. The red dashed line represents the mean of the distributions and the black vertical lines show the selected cutoff points.

The distribution of subjects across the groups was found to be imbalanced. The largest group was the Normal group with 782 examples, while other groups were smaller (AD: 178, V: 212, AV: 118, O: 90). In all groups the most extreme ages were under-represented, which rendered estimates at these extremes unreliable. To ensure reliable comparisons, it was deemed necessary to consider a minimum of five individuals over a two-year period. Consequently, a range was delineated for each group. In the context of a comparative analysis between two groups, only the area between the curves of the overlapped ranges of both groups was considered. Figure 2 illustrates variations in age distribution across groups and incorporates the designated cutoff points.

Moreover, the five study groups were divided by gender in order to analyze how gender affects the results. The gender distribution between the study groups is outlined in Table 1 along with their mean age. The majority of participants were approximately 70 years old, with a higher proportion of female participants (758) compared to male participants (622). The age distribution of both genders was comparable, with a higher proportion of women in the younger age group (50–60 years). Despite the average age being comparable between genders within a given group, it exhibited variation across different groups. The N group was observed to be the youngest, while the AV group was found to be the oldest.

**Table 1:** Number of examples per group and gender, their mean age and standard deviation, after preprocessing.

**Group**	**Women**		**Men**	
	**Examples (%)**	**Mean age (**σ**)**	**Examples (%)**	**Mean age (**σ**)**
N group	439 (56%)	62.8 (6.7)	343 (44%)	64.2 (6.8)
AD group	93 (52%)	68.4 (5.8)	85 (48%)	68.2 (6.7)
V group	115 (54%)	69.0 (6.5)	97 (46%)	69.5 (6.9)
AV group	62 (53%)	72.1 (5.1)	56 (47%)	73.2 (5.1)
OP group	49 (54%)	66.5 (5.4)	41 (45%)	71.0 (5.7)
TOTAL	758 (54%)	65.5 (7.2)	622 (45%)	66.9 (7.3)

### 2.4 Estimation of the volume of brain regions based on age

The present study analyses how the mean volume of each brain region changes with age. To this end, the Nadaraya-Watson kernel regression method (Nadaraya, 1964; Watson, 1964) was employed. This non-parametric technique is capable of identifying non-linear relations between two variables. In this study, the analyzed variables were age and volume, with the objective of obtaining a function that estimates the volume of each brain region for a given age. The function can be represented as a curve, which is referred to as the “volume curve”. This curve estimates the average volume of the brain region under study at differing ages. The application of kernel regression to diverse brain regions and study groups facilitated the analysis of disparities in brain aging between groups and brain regions.

In order to perform a robust curve estimation and assess the standard deviation of the computed mean curve at each point, 100 bootstrap curves were computed for each final curve in this study. The computation of each bootstrap curve was achieved by means of a sampling process in which samples were drawn with replacement, yielding a final sample of the same size as the original one. Consequently, the value and confidence of each point in the curve was estimated by the mean value and the standard deviation of the 100 points available.

The bandwidth, a parameter of the Nadaraya-Watson kernel regression model, is instrumental in determining the smoothness of the derived curve. Insufficient bandwidth can result in overfitted curves, whereas excess bandwidth can lead to uninformative, nearly constant curves. In order to calibrate the parameter for each study group and region, a cross-validation analysis was conducted. A preliminary visual analysis indicated that bandwidth values ranging from 5 to 15 were optimal for the data under consideration. In order to ascertain the most suitable bandwidth for each region, three curves were computed with different bandwidth values (5, 10, and 15) and the Mean Square Error metric, estimated by a 5-fold cross validation, was used to assess which bandwidth adapted best for the data points in each region and study group. All analyses were conducted utilizing the R version 4.5.1 statistical software environment.

### 2.5 Statistical significance of the difference between curves

A primary objective of this study was to make a comparison of the volume curves of the defined study groups. The comparison under scrutiny involved the computation of curve differences, which were determined by the area between the compared curves. Due to the smoothness of the curves, a resolution of one year was deemed adequate for this computation.

In order to assess the statistical significance of the computed differences, a test based on the chi-squared value was utilized, as outlined in the work of Hristova and Wimley (2023). In view of the nature of the study, which involved multiple curve comparisons (it should be noted that a separate curve was computed for each brain region and group), the p-values were adjusted for multiple tests using the Bonferroni correction. This conservative approach ensures a high degree of confidence when statistical differences are identified.

## 3 Results

The next subsections summarize first the differences of the volume curves measured as the area between two curves and then, the statistical significance analysis of the differences.

### 3.1 Qualitative analysis of differences

The primary objective of the analysis was to ascertain whether the results for the Normal group were influenced by the inclusion of cognitively normal subjects who exhibited early indicators of pathology. To this end, the volume curves of the N group and the entire database were computed, and the area between them was then used to establish a ranking of brain regions according to their level of difference.

Although the N group was included in the complete database and was the predominant group, disparities in brain region volume curves between the N group and the entire dataset becoae evident. Table 2 (first column) presents the ten regions that exhibited the largest area between the curves. The regions that exhibited the most significant disparities were specifically the entorhinal areas and the amygdalas.

**Table 2:** Top 10 regions with the largest area between curves in the comparison between the N group and the complete dataset. The results obtained for both genders are also included.

**Complete dataset**		**Men**		**Women**	
**Region**	**Diff**.	**Region**	**Diff**.	**Region**	**Diff**.
LLENTAV	4.78	LLAMYG	7.48	LLAMYG	4.91
LLAMYG	4.45	LLENTAV	7.16	LLPUTV	4.61
LRAMYG	4.04	LRAMYG	6.98	LLPLANPV	4.49
LRENTAV	3.99	LRENTAV	6.50	LRBFBV	4.34
LRMTGV	3.77	LRINFOGV	5.72	LRAMYG	4.30
LLILVV	3.68	LLOCFUGV	5.54	LCVLVIIIX	3.99
LRBFBV	3.54	LRCOPERV	5.13	LMEDUV	3.84
LRINFOGV	3.52	LLMOCCGV	5.12	LRMOCCGV	3.73
LRPOSTGV	3.52	LRBFBV	4.76	LLMFCV	3.79
LLPLANPV	3.51	LLILVV	4.71	LRENTAV	3.73

Abbreviations correspond to the following regions: LCVLVIIIX, Cerebellar Vermal Lobules Viii-X; LLAMYG, Left Amygdala; LLENTAV, Left Entorhinal Area; LLILVV, Left Inferior Lateral Ventricle; LLMFCV, Left Medial Frontal Cortex; LLMOCCGV, Left Middle Occipital Gyrus; LLOCFUGV, Left Occipital Fusiform Gyrus; LLPLANPV, Left Planum Polare; LLPUTV, Left Putamen; LMEDUV, Medulla; LRAMYG, Right Amygdala; LRBFBV, Right Basal Forebrain; LRCOPERV, Right Central Operculum; LRENTAV, Right Entorhinal Area; LRINFOGV, Right Inferior Occipital Gyrus; LRMOCCGV, Right Middle Occipital Gyrus; LRMTGV, Right Occipital Pole; LRPOSTGV, Right Postcentral Gyrus Medial Segment.

**Figure 3:**
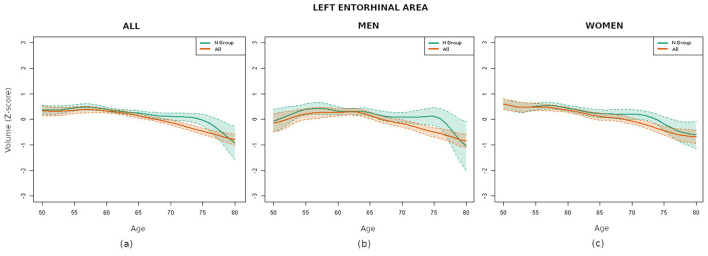
Comparison of the volume curves of the left entorhinal area for the N group (green) and all participants (orange). **(a)** Curves corresponding to all the subjects analyzed. **(b)** Curves corresponding to the male subjects. **(c)** Curves corresponding to the female subjects.

In order to ascertain whether the results obtained were consistent across both genders, the experiment was repeated with the database being divided into two subsets: one comprising male subjects and the other comprising female subjects. It is interesting to note that, as illustrated in Table 2 (central and right columns), a more pronounced discrepancy was evident between the curves for male subjects in comparison to those for female subjects. Furthermore, the regions previously identified as the most divergent maintained their distinction in the male group, while the entorhinal area no longer featured among the most disparate regions in the female subject group. As an illustrative example, Figure 3 shows the curves of the left entorhinal area.

**Figure 4:**
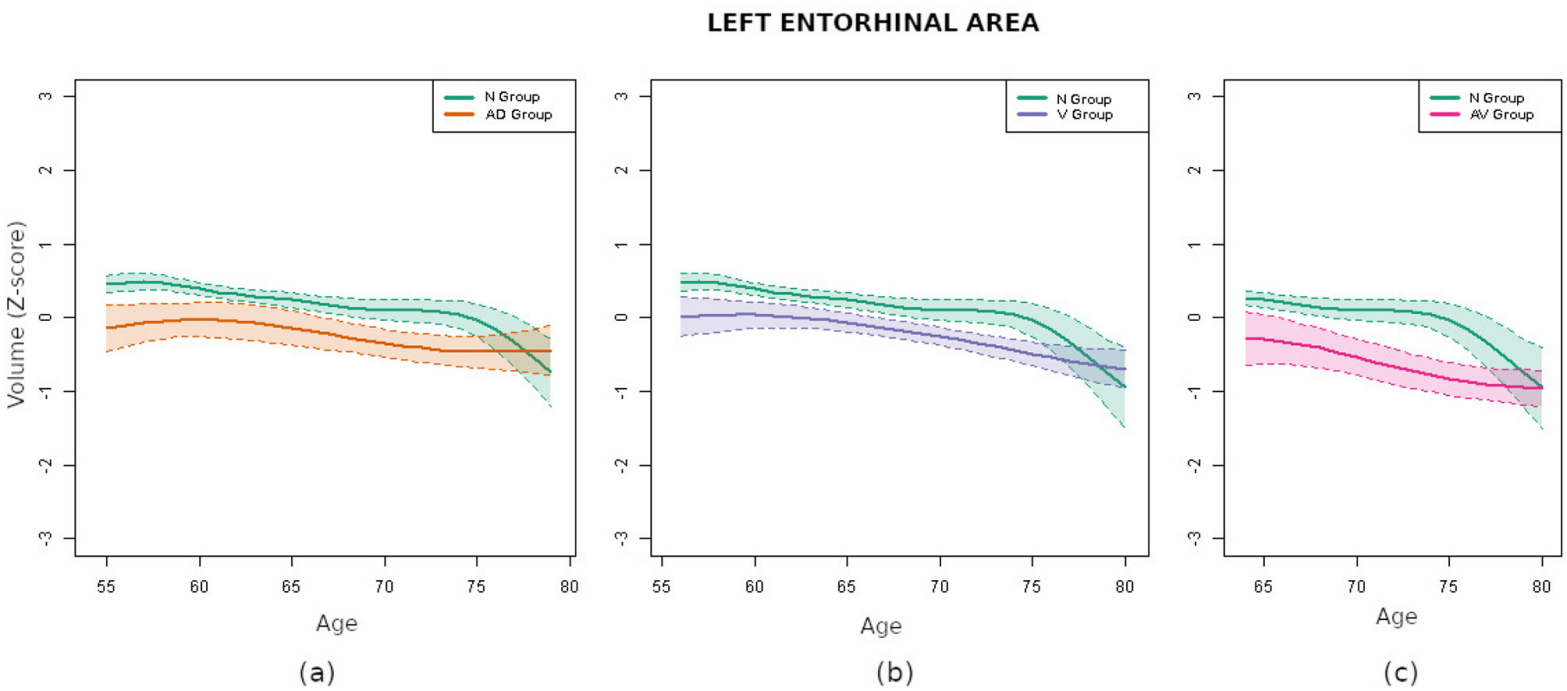
Comparison of the volume curves of the left entorhinal area for the N group (green) and the pathological groups. **(a)** Comparison corresponding to the AD group (orange). **(b)** Comparison corresponding to the V group (purple). **(c)** Comparison corresponding to the AV group (pink).

When comparisons were carried out between the curves of the N group and those obtained for individuals with biomarkers—AD group, V group and AV group—the differences were, as expected, more pronounced. In particular, as Table 3 shows, the areas between the most different curves exhibited an approximate increment of 100%. A close examination revealed some parallels between the results observed in the AD group and those seen in the AV group. This observation was not unexpected, given the presence of shared markers between these two groups. Notably, both amygdalas and the left entorhinal area exhibited the most significant differences. In contrast, when contrasting the N and V groups, the regions that differed the most were significantly different. This observation may suggest that distinct forms of brain deterioration are caused by different pathologies. In particular, the left entorhinal area exhibited the most pronounced differences, while the amygdalas did not show significant variations. For the purpose of visual assessment, Figure 4 presents the curves corresponding to the left entorhinal area, a region that demonstrated significant disparities in the three aforementioned comparisons.

**Table 3:** Top 10 regions with the largest area between curves in the comparison between the Normal group and the pathology groups.

**Alzheimer (AD)**		**Vascular (V)**		**Alzh. + Vasc. (AV)**		**Others (OP)**	
**Region**	**Diff**.	**Region**	**Diff**.	**Region**	**Diff**.	**Region**	**Diff**.
LRAMYG	10.36	LLENTAV	8.41	LLAMYG	9.71	LVV	11.60
LLENTAV	10.10	LRCV	7.97	LLENTAV	9.65	LCVLVIVII	11.37
LLAMYG	9.98	LRFOPV	7.63	LRAMYG	9.03	LCVLVIVV	10.11
LRENTAV	9.24	LROPIFGV	7.47	LLPAHIPGV	8.98	LRTHAPV	9.28
LHVL	8.04	LRPOSTGV	7.30	LRMTGV	8.89	LTVV	8.95
LHVR	7.97	LRBFBV	7.24	LRENTAV	8.44	LRSUPMCV	8.87
LRMTGV	7.34	LLCV	7.10	LLSTPGV	8.42	LLPOBGV	8.69
LLANGV	7.00	LLPAHIPGV	6.96	LHVL	8.21	LRCBEV	8.59
LLILVV	6.54	LLOCFUGV	6.94	LLPRECV	7.78	LRCALCV	8.45
LLACCU	6.28	LTVV	6.80	LLILVV	7.77	LLSFGV	8.29

Abbreviations correspond to the following regions: LCVLVIVII, Cerebellar Vermal Lobules Vi-Vii; LCVLVIVV, Cerebellar Vermal Lobules I-V; LHVL, Left Hippocampus; LHVR, Right Hippocampus; LLACCU, Left Accumbens Area; LLAMYG, Left Amygdala; LLANGV, Left Angular Gyrus; LLILVV, Left Inferior Lateral Ventricle; LLCV, Left Caudate; LLENTAV, Left Entorhinal Area; LLPAHIPGV, Left Parahippocampal Gyrus; LLPOBGV, Left Posterior Orbital Gyrus; LLPRECV, Left Precuneus; LLSFGV, Left Superior Frontal Gyrus; LLSTPGV, Left Superior Temporal Gyrus; LRAMYG, Right Amygdala; LRBFBV, Right Basal Forebrain; LRCALCV, Right Calcarine Cortex; LRCBEV, Right Cerebellum Exterior; LRCV, Right Caudate; LRENTAV, Right Entorhinal Area; LRFOPV, Right Frontal Operculum; LRMTGV, Right Occipital Pole; LROPIFGV, Right Opercular Part of The Inferior Frontal Gyrus; LRPOSTGV, Right Postcentral Gyrus Medial Segment; LRSUPMCV, Right Supplementary Motor Cortex; LRTHAPV, Right Thalamus Proper; LTVV, Third Ventricle; LVV, Ventricular.

### 3.2 Significance analysis of differences

**Figure 5:**
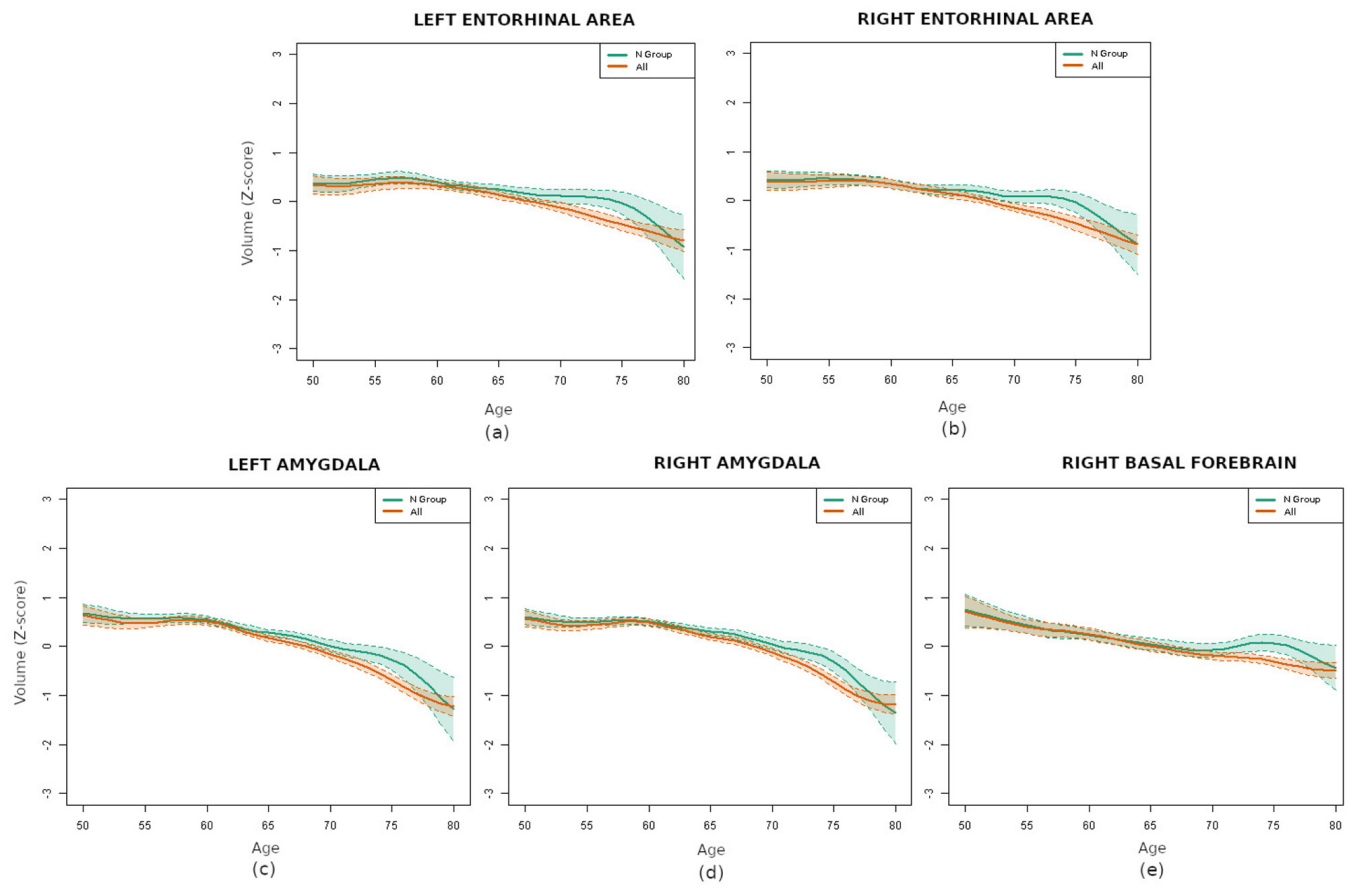
Comparison of the volume curves for the regions with statistically significant differences between the N group (green) and the whole database (orange): **(a)** left entorhinal area, **(b)** right entorhinal area, **(c)** left amygdala, **(d)** right amygdala, and **(e)** right basal forebrain.

The subsequent significance analysis revealed that five regions exhibited statistically significant differences (p < 0.01) between the N group and the entire database. Remind that the p-values were adjusted for multiple comparisons with the Bonferroni correction, which is highly conservative. These regions were both entorhinal areas, both amygdalas and the right basal forebrain, whose volume curves are depicted in Figure 5. Therefore, even if a representative sample contained fewer pathological subjects than normal ones, its effects on specific brain regions were pronounced enough to affect their curves. This result confirmed the importance of strict filtering criteria in brain aging related studies.

Conducting the analysis separately for each gender requires the utilization of smaller samples, thereby increasing the difficulty in identifying statistically significant differences. Still, the results indicated statistically significant differences in the right amygdala in women and in the left amygdala and right entorhinal area in men.

The comparison of the N group with the pathological groups (AD, V and AV) revealed that numerous regions exhibited remarkably small p-values. This finding suggests that the differences in brain volume between the healthy and pathological groups were highly significant. Specifically, the differences were found to be statistically significant (p-value < 0.01, adjusted for multiple tests with Bonferroni) for 27 regions in the AD group, for 61 in the V group and for 64 in the AV group. The five regions that demonstrated significant differences when comparing the N group with the entire dataset were present in all cases, with the exception of the right amygdala, which was absent in the V group. For a comprehensive set of results, refer to the Supplementary material.

## 4 Discussion

In this cross-sectional study of cognitively unimpaired adults, we estimated smoothed age-volume associations for cortical and subcortical regions and quantified between-group separations as the area between curves. Three regions showed the most consistent and robust separations when comparing biomarker-negative participants with pathology-enriched profiles: the entorhinal cortex, bilateral amygdalae, and the right basal forebrain (significant in the pooled sample after Bonferroni correction). Contrasts with AD-enriched (A+V–) and combined AD+vascular (A+V+) groups displayed a medial temporal emphasis (entorhinal and amygdala), consistent with classic staging and modern *in-vivo* literature showing early entorhinal vulnerability and prominent amygdalar involvement. In contrast, the vascular (V+A–T–) group exhibited a distinct, fronto-subcortical/opercular pattern with limited amygdalar involvement. This framework aligns with contemporary diagnostic revisions and provides biological context for the topographies observed in our subgroup contrasts (Jack et al., 2024; Dubois et al., 2024).

The entorhinal cortex is consistently reported as one of the first regions to show Alzheimer-related pathology. Postmortem and in-vivo work indicates early neurofibrillary changes and neuronal loss—particularly in layer II—well before symptoms (Braak and Braak, 1991, 1995; Gómez-Isla et al., 1996). Imaging studies detect entorhinal cortex atrophy in preclinical and prodromal stages and link it to subsequent cognitive decline. Given the role of the entorhinal cortex as a hub between hippocampus and neocortex—-supporting memory and spatial navigation—early dysfunction has disproportionate clinical impact (Khan et al., 2014; Igarashi, 2023; Karimani et al., 2024). In our cross-sectional analyses, entorhinal differences were especially marked in AD-enriched groups, consistent with its utility as a structural biomarker for early detection and monitoring (Velayudhan et al., 2013). On the other hand, it is increasingly recognized that vascular pathology can exacerbate neurodegeneration in the medial temporal circuits (Arvanitakis et al., 2016). Studies showed that the coexistence of small vessel disease (SVD) or white matter hyperintensities (WMH) with AD pathology is associated with greater atrophy and a more rapid cognitive decline (Debette et al., 2019; van der Flier et al., 2018). These findings support the notion that vascular injury and AD pathology may act synergistically in this region, as observed in our combined A+V+ subgroup.

Placing this result in an age-sensitive context, the entorhinal divergence we observed beginning around age 55 is supported by prior work showing that structural change in the medial temporal lobe emerges by midlife and is already detectable in the late 50s (Thomann et al., 2013). Cross-sectional morphometry in cognitively normal adults demonstrates significant entorhinal cortex shrinkage when comparing cohorts aged 53–55 vs. 73–75 years, indicating an age-sensitive trajectory well before overt symptoms (Thomann et al., 2013). Lifespan data further show that entorhinal thickness peaks in the mid-40s and subsequently declines, making entorhinal volume differences expected by ages 55–60 (Hasan et al., 2015). Longitudinal MRI confirms age-related entorhinal cortex atrophy rates in cognitively normal elders (Du et al., 2006). In parallel, tau-PET studies in clinically normal individuals reveal early medial temporal tau (Braak I/II) that increases with age most prominently in entorhinal-cortex and relates to cortical thinning and subtle memory change (Jack et al., 2018; Knopman et al., 2019), providing a mechanistic substrate for early entorhinal cortex volumetric separation between biomarker-negative and biomarker-positive groups.

The amygdala has gained renewed attention for early involvement along the AD continuum. Our findings, in line with MRI and neuropathology studies, suggest amygdalar volume differences in AD-enriched contrasts (Poulin et al., 2011; Salman et al., 2024). Although we did not examine specific amygdalar subnuclei, a previous study reported greater vulnerability in certain regions, such as the basolateral complex, potentially linked to both early cognitive changes and neuropsychiatric symptoms (Padulo et al., 2023). Taken together, these findings support the relevance of including amygdala volume as a potentially early marker of AD. However, it remains important to determine whether this atrophy is specific to Alzheimer's disease or also is present in other neurodegenerative conditions. For example, previous studies suggested an association between vascular risk factors and amygdala volume loss (Habes et al., 2016; Prins and Scheltens, 2015). In contrast, our data did not reveal significant differences between the normal group and the vascular pathology group after the multiple-comparison correction.

The basal forebrain, and particularly the nucleus basalis of Meynert, plays a fundamental role in providing cholinergic input to the cerebral cortex. Its early vulnerability in the course of Alzheimer's disease was well documented, with some studies suggesting that degeneration in this area might even precede cortical atrophy (Grothe et al., 2015; Schmitz et al., 2016). In our sample, we also detected volumetric reductions in this region among individuals with biomarker positivity. This supports its potential utility as a sensitive indicator of early neurodegeneration. However, whether these structural changes can reliably predict cognitive decline remains an open question, and future research should focus on validating its prognostic value in longitudinal settings. Emerging evidence suggests that vascular pathology can negatively impact basal forebrain integrity. For example, white matter lesions and microvascular disease were associated with reduced basal forebrain volume and impaired cholinergic function, which may amplify cognitive deficits in the presence of AD pathology (Schmitz et al., 2016; Korte et al., 2020). This interaction may explain the pronounced atrophy observed in our mixed pathology group.

Compared with the Normal group, the vascular-enriched subgroup showed a distinc fronto-subcortical/opercular pattern with somatosensory involvement (e.g., caudate, frontal/opercular territories, and postcentral regions) whereas amygdalar differences did not remain significant after the multiple-comparison correction. This dissociation is consistent with SVD's preferential impact on fronto-subcortical networks via white-matter pathway disruption shown in structural/functional network studies (Lawrence et al., 2014). White-matter hyperintensities and microbleeds disrupt long-range association fibers and basal ganglia-thalamo-cortical loops, yielding cortical thinning and subcortical volume alterations through disconnection cascades (Zanon Zotin et al., 2021). Longitudinal evidence in cognitively unimpaired cohorts shows a mutually reinforcing relationship between WMH progression and cortical thinning, indicating entanglement before detectable cognitive deficits—fully aligned with the broader spatial footprint we observed in V+ (Bernal et al., 2024). The anatomical distribution of microbleeds further supports etiology-specific effects: strictly lobar (often with cerebellar involvement) is characteristic of cerebral amyloid angiopathy (CAA), whereas deep/infratentorial microbleeds are more typical of hypertensive arteriolosclerosis or mixed etiologies (Jung et al., 2020). In addition, periventricular vs deep WMH likely reflect partly distinct pathobiologies/age-related processes, reinforcing the need to consider Fazekas PWMH/DWMH compartments when modeling SVD burden (Pradeep et al., 2024).

## 5 Conclusions

Overall, these results support biomarker-informed stratification (A/T/N and vascular burden) to avoid bias in cross-sectional age-volume associations among cognitively unimpaired adults. The patterns observed, medial temporal in AD-enriched and fronto-subcortical/opercular in vascular profiles, are consistent with established neuropathological models.

Clinically, these results emphasize the need to focus monitoring and early intervention efforts on individuals showing volumetric alterations in these regions, potentially delaying or preventing symptom onset.

## 6 Limitations

While the present study offers valuable insights into early brain changes linked to Alzheimer's disease and vascular pathology, it is important to acknowledge some inherent limitations. Firstly, the cross-sectional nature of the data means that we are capturing a snapshot in time rather than following individuals as their brains change over the years. All curves depict smoothed cross-sectional age-volume associations and, thus, they do not imply within-person longitudinal change. This limits our ability to draw firm conclusions about cause and effect or to predict how these brain changes might evolve in the future. Longitudinal studies, which involve tracking the same individuals over time, will be essential to confirm whether the observed patterns truly forecast cognitive decline or disease progression.

Additionally, although we carefully stratified participants based on biomarker profiles, the groups exhibiting vascular and Alzheimer's pathologies were comparatively smaller in size than the Normal group. This imbalance might reduce the statistical power to detect subtler differences or to fully capture the diversity present within these pathological groups. We did not perform formal pairwise tests among AD, V, and AV groups; therefore, any apparent between-pathology differences should be considered hypothesis-generating and will require adequately powered confirmatory analyses. We also recognize that factors like lifestyle, education, or other health conditions—which can influence brain structure and resilience—were not fully accounted for in this analysis.

Furthermore, although contemporary criteria and longitudinal evidence indicate that tau positivity (T+) is most tightly coupled to neurodegeneration in cognitively unimpaired cohorts, our sample size did not permit T-stratified analyses within the A+ group; therefore, A+T– and A+T+ were analyzed together. This pooling likely blunts regional separations relative to what would be expected in A+T+ specifically, and we anticipate larger effect sizes in T-stratified (A+T– vs. A+T+) analyses when adequately powered.

Finally, while advanced imaging and statistical methods allowed us to detect meaningful differences, no single biomarker or brain region can tell the whole story. Brain aging and neurodegeneration are complex processes influenced by many interacting factors. Therefore, our findings should be interpreted as part of a broader puzzle that requires integration with clinical, genetic, and other biological data to truly understand and intervene effectively.

## Data Availability

The data analyzed in this study is subject to the following licenses/restrictions: Data is available upon request approval in AD (Alzheimer Disease Data Initiative) workbench. Requests to access these datasets should be directed to The European Prevention of Alzheimer's Dementia Consortium: https://ep-ad.org/.
